# A type of anteroseptal left ventricular ballooning in a patient with Takotsubo cardiomyopathy

**DOI:** 10.1007/s12471-017-1056-2

**Published:** 2017-11-08

**Authors:** K. Goto, S. Kasama, M. Kurabayashi

**Affiliations:** 1 Division of Cardiology, Isesaki Municipal Hospital, Isesaki, Gunma Japan; 2 Department of Medicine and Biological Science, Gunma University Graduate School of Medicine, Maebashi, Japan

**Fig. 1:**
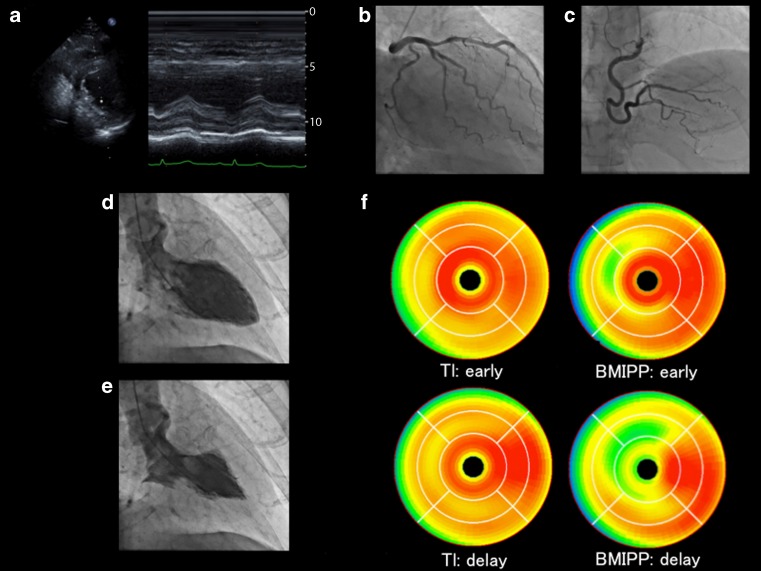
**a** Transthoracic echocardiogram on arrival showing akinesia of the anteroseptal wall, **b** and** c** Emergency coronary angiograms. Neither the left coronary artery nor the right coronary had occlusion or significant stenosis. **d** and **e** Left ventriculograms. The mid-left ventricle was severely hypokinetic. **f** Single photon-emission computed tomogram, polar and heart score view maps of thallium myocardial perfusion and BMIPP images. *BMIPP* iodine-123-labeled 15-(p-iodophenyl)-3-R′S‐methylpentadecanoic acid, *TI* thallium 201

A 62-year-old man presented to our hospital with chest pain. The electrocardiogram revealed ST-segment elevation in leads V1 through V3. The transthoracic echocardiogram showed akinesia of the anteroseptal wall (Fig. 1a). The coronary angiogram (Fig. 1b, c) revealed no significant stenosis or occlusion in the coronary artery. However, the left ventriculogram showed severe hypokinesis of the mid-left ventricle, while the basal left ventricle and left ventricular apex were hyperkinetic (Fig. 1d, e). The thallium-201 scintigram (Fig. 1f, left upper and lower images) and iodine-123-labeled 15-(p-iodophenyl)-3-R′S-methylpentadecanoic acid (BMIPP) (Fig. 1f, right upper and lower images) showed myocardial ischaemia in the anteroseptal wall, regardless of normal coronary blood flow. We diagnosed him as anteroseptal left ventricular ballooning with Takotsubo cardiomyopathy.

Takotsubo cardiomyopathy has been reported previously. However, the present case was rare with respect to the anteroseptal left ventricular ballooning.

